# Application of α-bromination reaction on acetophenone derivatives in experimental teaching: a chemical innovation experiment engaging junior undergraduates

**DOI:** 10.1186/s13065-024-01145-y

**Published:** 2024-02-21

**Authors:** Yongguang Gao, Na Chen, Xiaoye Jiang, Xiaochun Yang

**Affiliations:** Department of Chemistry, Tangshan Normal University, Tangshan, China

**Keywords:** Acetophenone, Pyridine hydrobromide perbromide, Acetic acid, α-Substitution, Experimental pedagogy

## Abstract

The α-bromination reaction of carbonyl compounds is a significant topic in the field of organic chemistry. However, due to the lack of suitable brominating reagents, the application of this reaction in undergraduate organic chemistry experiments is limited. In this study, three junior undergraduates successfully conducted an innovative experiment under the guidance of teachers. The bromination of various acetophenone derivatives was investigated by employing pyridine hydrobromide perbromide as the brominating agent, with a focus on exploring the effects of reaction time, reaction temperature, and dosage of the brominating agent. The results demonstrated that 4-chloro-α-bromo-acetophenone could be synthesized at 90 ℃ using 4-chloroacetophenone as a substrate and acetic acid as a solvent with a molar ratio of substrate to brominator being 1.0:1.1. Through the experimental teaching of 18 junior undergraduates, it was observed that all the students successfully completed the experiment within a time frame of 4–5 h, with a notable achievement yield exceeding 80% observed in 14 students. This innovative experiment exhibits significant advantages in terms of safety, high yield, cost-effectiveness, and repeatability. Furthermore, while reinforcing fundamental skills in chemistry experimentation among students, it enhances their scientific literacy levels and fosters innovation consciousness as well as practical aptitude. Consequently, this approach is highly suitable for widespread implementation and integration into undergraduate experimental pedagogy.

**Supplementary Information:**

The online version contains supplementary material available at 10.1186/s13065-024-01145-y.

## Introduction

The organic chemistry experiment is a fundamental course for students majoring in chemistry, medicine, pharmacy, and biology [1–4]. It plays an essential role in the teaching of organic chemistry as most theories and laws are derived from experimental findings. Moreover, the application and evaluation of these theories should be based on exploration and testing through experiments. Incorporating important research results into undergraduate experimental teaching not only consolidates their theoretical knowledge but also deepens their understanding of organic reaction principles while cultivating scientific literacy, research ability, innovation ability, and timely awareness of international frontier trends - all crucial tasks for educators today [5–7].

The bromination reaction of carbonyl compounds is a crucial aspect of organic chemistry. α-Brominated products derived from bromoacetophenone are significant intermediates in organic synthesis and find extensive applications in the production of pharmaceuticals, pesticides, and other chemicals [8–10]. For instance, α-bromoacetophenone is a significant intermediate for non-steroidal anti-inflammatory drug aryl propionate [11], while *p*-methoxy-α-bromoacetophenone acts as the primary intermediate in synthesizing the estrogenic drug raloxifene [12, 13]. O-chloro-alpha-bromoacetophenone plays a vital role as an intermediate for clorprenaline [14]. These derivatives are typically obtained through α-bromination of acetophenone derivatives using brominating agents. Commonly used bromination reagents include liquid bromine, *N*-bromosuccinimide (NBS), and copper bromide [15, 16]. However, these reagents pose several challenges when applied to undergraduate experiments. Liquid bromine exhibits high toxicity, strong corrosiveness, low reaction selectivity, environmental pollution risks and poor safety measures. NBS exhibits poor thermal stability due to its incompatibility with common solvents such as tetrahydrofuran, DMF, and toluene, leading to autocatalytic decomposition [17]. Although copper bromide offers high safety and good thermal stability, it contains heavy metal copper ions which contradict the principles of green chemistry development and render it unsuitable for application in chemical experiment teaching. Therefore, the development of safe, efficient and green bromine reagents and their application in the substitution reaction of acetophenone derivatives is of great significance to expand the scope of undergraduate chemistry teaching experiments. In this study, pyridine hydrobromide perbromide was chosen as the preferred bromination reagent with optimized conditions including reaction substrate selection along with reaction time and temperature adjustments aiming to identify suitable reaction conditions applicable for promoting chemistry experiment teaching among undergraduates.

## Pedagogical goals and assessment

**Scheme 1:**
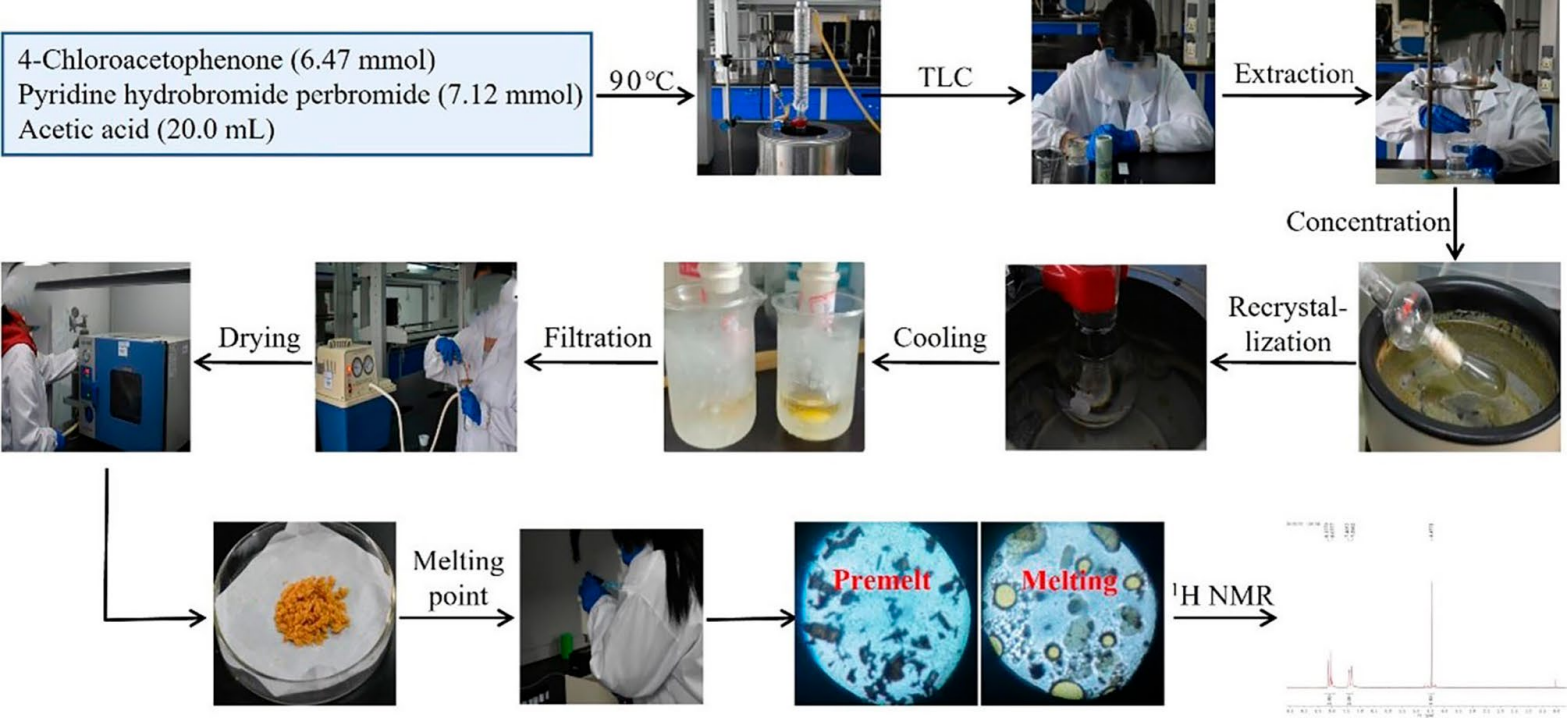
Schematic illustration of synthesis of α-bromoacetophenone derivatives

“Chemical Innovation Experiment” is a significant elective course established by Tangshan Normal College for third-year college students, aiming to foster their innovative consciousness, entrepreneurial spirit, and innovative abilities. The objective of this experimental innovation project is to design fundamental experiments that reflect novel knowledge, theories, methods, and technologies from both domestic and international scientific research results, catering specifically to the needs of undergraduate teaching. Students are required to consult relevant literature and chemistry textbooks to identify suitable topics which they will then discuss with their teachers before completing them under their guidance. This experimental endeavor was accomplished by three students over a span of four months under the guidance of teachers during weekends and spare time. The selection of topics, route design, and exploration of conditions were collaboratively completed by the three students. The specific contents are as follows: (a) “Why is there no synthesis of α-bromoacetophenone derivatives in Organic Chemistry Experiment?” (Questioning); (b) Commonly used bromination reagents are either volatile, toxic or expensive, rendering them unsuitable for undergraduate experimental teaching (Problem analysis); (c) Seeking bromination reagents with high safety and low cost while utilizing scientific research methods to explore experimental conditions suitable for application in teaching experiments (Problem-solving). Scheme 1 illustrates the process undertaken by the students during their exploration. The experimental innovation project spanned a duration of approximately four months. Subsequent to its completion, the three participating students were requested to document their experimental experiences (Figure S1). Following an arduous four-month endeavor, their awareness of innovation, aptitude for scientific research, and proficiency in teamwork exhibits significant enhancement. Furthermore, during weekly group meetings, they exhibited an enhanced desire to share ideas and communicate with their teacher compared to before the experiment; their curiosity towards exploring uncharted territories has also intensified.

We recruited 18 junior college students to assess the optimized synthesis method of 2-bromo-4’-chloro-acetophenone through innovative experiments (Experimental scheme can be found in the supporting materials), encompassing experimental procedures, reports, and evaluations. To enhance the difficulty level during experimental teaching, each student independently conducted experiments involving device setup, reaction process monitoring, and product separation and purification. For real-time visualization of experimental teaching and students’ monitored reaction progress using TLC, refer to supporting Figure S2. The experimental results demonstrated that all students successfully obtained the desired product (2-bromo-4’-chlorophenone) within a timeframe of 4–5 h. Table 1 presents an analysis of reaction yields; out of the 18 participants, 14 achieved yields exceeding 80%, with only one student producing less than 60%. Consequently, this experiment is deemed suitable for implementation in undergraduate chemistry laboratory instruction.

**Table 1:** Record of students’ experimental reports

Yield	< 60%	61–70%	71–80%	81–90%	> 90%
Number of students	1	1	2	11	3
Percentage	5.6%	5.6%	11.1%	61.1%	16.7%

## Experiment

### Experimental principle

**Fig. 1:**
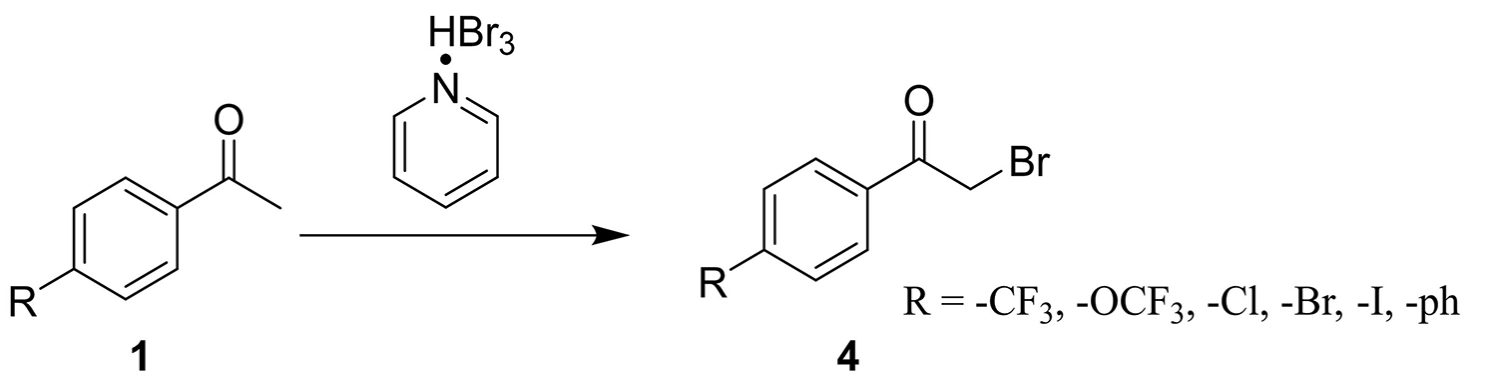
The reaction route of α-bromination of acetophenone derivatives

In this experiment, α-bromoacetophenone derivatives (4-trifluoromethyacetophenone, 4-trifluoromethoxyacetophenone, 4-chloroacetophenone, 4-bromoacetophenone, 4-iodoacetophenone and 4-phenylacetophenone) were employed as starting materials. Pyridine hydrobromide perbromide (pyridinium tribromide) was utilized as the brominating agent for the synthesis of α-bromoacetophenone derivatives (Fig. 1). The impact of the brominating agent on the substitution reaction of various acetophenone derivatives under different conditions was investigated.

**Fig. 2:**
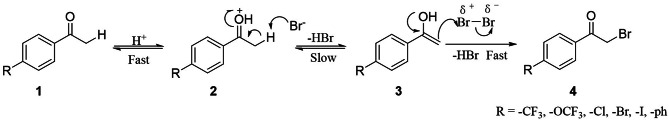
The reaction mechanism of α-bromination of acetophenone derivatives

The reaction mechanism of α-bromination of acetophenone derivatives is illustrated in Fig. 2 [18]. Under acidic conditions, acetophenone derivative **1** undergoes protonation to yield protonated carbonyl compound **2**. Subsequently, bromine anions attack the hydrogen atoms on the alpha carbon of compound **2**, leading to the formation of enolate product **3** after debromination. Compound **3** then undergoes a nucleophilic attack by the electron on positively charged bromine ions, resulting in the formation of compound **4** through the creation of a carbon-oxygen double bond with the lone electron pair on hydroxyl oxygen. The rate-determining step for this reaction is identified as the second step involving enol form **3**. In cases where an electron-donating group is attached to the alpha carbon of the carbonyl compound, it becomes challenging for protons to leave and consequently slows down the reaction rate. Conversely, when an electron-withdrawing group is present at this position, protons are more easily released and thus accelerate the reaction rate accordingly. Similarly, attachment of an electron-withdrawing group to a benzene ring also facilitates α-bromination reactions in acetophenone derivatives. Compared with α-carbon substituted derivatives of acetophenone, those substituted with electron-withdrawing groups on benzene rings exhibit lower costs. Therefore, we selected six different substrates consisting of acetophenone derivatives substituted with various electron-withdrawing groups to investigate how pyridine hydrobromide perbromide affects α-bromination reactions.

### Instruments and reagents

**Fig. 3:**
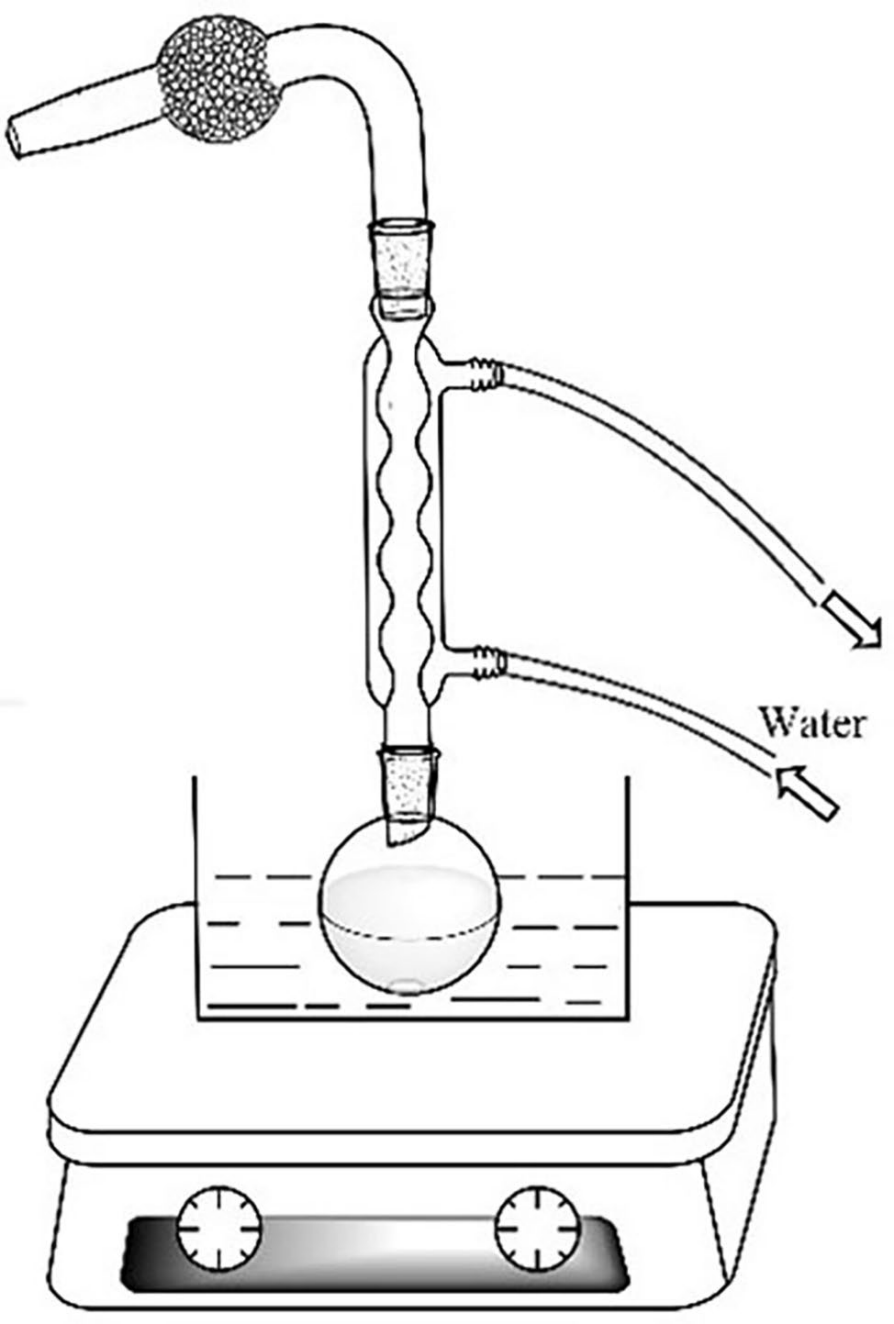
The reaction device of α-bromination of acetophenone derivatives

The main reagents and instruments necessary for the experiment are presented in Table S1 and Table S2, respectively. Additional apparatus includes a magneton, iron frame, Brinell funnel, glass rod, 50 mL round-bottom flask, 100 mL conical flask, 100 mL measuring cylinder, and a 150 mL liquid separation funnel. The reaction device is shown in Fig. 3. The drying tube, located at the upper extremity of the condenser, is charged with anhydrous calcium chloride.

### Experimental procedure

The synthesis process of α-bromoacetophenone derivatives is demonstrated using 4-chloroacetophenone as a representative example. Other α-bromoacetophenone derivatives in this study were synthesized following the same procedure. 4-Chloroacetophenone (0.77 g, 5.0 mmol), pyridine hydrobromide perbromide (1.76 g, 5.5 mmol), and acetic acid (20 mL) were combined in a 50 mL round-bottom flask equipped with a condensing tube, and the reaction mixture is stirred at 90 ^o^C. The reaction progress was monitored via thin layer chromatography (TLC), and the depletion of the starting material was observed at a time point of 3 h post-reaction. Subsequently, the reaction mixture was poured into an ice water bath (50 mL) and extracted twice with ethyl acetate (20 mL × 2). Organic phase was sequentially washed with saturated sodium carbonate solution (30 mL) and saturated saline solution (30 mL). After drying over anhydrous sodium sulfate, the solvent was removed under reduced pressure using a rotary evaporator to afford crude solid product which underwent recrystallization from petroleum ether (-4 ^o^C). The resulting orange solid product weighed 0.97 g with a yield of 83%. The recrystallized product’s identity was confirmed by ^1^H NMR spectroscopy, while its melting point value matched that reported in literature.

## Results and discussion

### Effect of material ratio on yield of α-bromoacetophenone derivatives

Using acetic acid as the solvent, we investigated the impact of the substrate to bromine reagent ratio on the reaction yield of 4-trifluoromethyacetophenone, 4-trifluoromethoxyacetophenone, 4-chlorophenone, 4-bromoacetophenone, 4-iodoacetophenone, and 4-phenylacetophenone with pyridine hydrobromide perbromide at 90 ^o^C. As presented in Table 2, when maintaining a substrate to bromine reagent ratio of 1.0:1.1, the reaction yield exceeded 66%. Among these six substrates examined, 4-trifluoromethylacetophenone exhibited superior electron absorption ability and achieved the highest yield (90%).

**Table 2:** Effects of material ratio on yield of acetophenone derivatives

Molar Ratio	1.0 : 0.8	1.0 : 1.0	1.0 : 1.1
4-Trifluoromethylacetophenone (%)	82 ± 7	88 ± 4	90 ± 5
4-Trifluoromethoxyacetophenone (%)	73 ± 3	86 ± 4	88 ± 6
4-Chloroacetophenone (%)	62 ± 5	80 ± 7	85 ± 4
4-Bromoacetophenone (%)	60 ± 6	69 ± 5	78 ± 4
4-Iodoacetophenone (%)	43 ± 3	55 ± 4	66 ± 5
4-Phenylacetophenone (%)	45 ± 5	58 ± 4	70 ± 4

### Effect of temperature on yield of α-bromoacetophenone derivatives

Under the molar ratio of n(_acetophenone derivative)_:n_(pyridine hydrobromide perbromide)_ = 1.0:1.1, we investigated the impact of reaction temperature on the substitution reaction of acetophenone derivative. As depicted in Table 3, when the reaction temperature is below 80 ^o^C, the bromination yield of acetophenone derivatives is relatively low. However, when the reaction temperature reaches 90 ^o^C, its impact on the yield becomes insignificant. With the increase of temperature, the yield decreases slightly, which may be attributed to the formation of dibromine substitution products. The objective of our study is to achieve high-yield bromination while minimizing the reaction temperature, thus making 90 °C the optimal choice for this reaction.

**Table 3:** Effects of reaction temperature on yield of acetophenone derivatives

Temperature /℃	80	90	100	120
4-Trifluoromethylacetophenone (%)	85 ± 8	90 ± 5	88 ± 6	87 ± 5
4-Trifluoromethoxyacetophenone (%)	80 ± 6	88 ± 6	86 ± 5	85 ± 4
4-Chloroacetophenone (%)	74 ± 6	85 ± 4	82 ± 4	83 ± 3
4-Bromoacetophenone (%)	65 ± 4	78 ± 4	80 ± 6	76 ± 4
4-Iodoacetophenone (%)	50 ± 4	66 ± 5	70 ± 5	72 ± 5
4-Phenylacetophenone (%)	55 ± 5	70 ± 4	72 ± 5	76 ± 6

### Effect of reaction time on yield of acetophenone derivatives

The reaction time for undergraduate organic chemistry experiments should be 4-hour timeframe. Therefore, we investigated the yield of α-bromoacetophenone derivatives at three specific time intervals: 2, 3, and 4 h. Table 4 revealed that when the ratio of acetophenone derivative to pyridine hydrobromide perbromide is maintained at 1.0:1.1 under a temperature of 90 °C, the highest yield was obtained after a reaction duration of precisely 3 h. However, as the reaction time increases beyond this point, there is a gradual decrease in yield accompanied by an increase in undesired by-products.

**Table 4:** Effects of reaction time on yield of acetophenone derivatives

Reaction Time/h	2	3	4
4-Trifluoromethylacetophenone (%)	85 ± 5	90 ± 5	86 ± 4
4-Trifluoromethoxyacetophenone (%)	84 ± 6	88 ± 6	86 ± 5
4-Chloroacetophenone (%)	82 ± 5	85 ± 4	84 ± 3
4-Bromoacetophenone (%)	73 ± 4	78 ± 4	76 ± 2
4-Iodoacetophenone (%)	62 ± 3	66 ± 5	68 ± 6
4-Phenylacetophenone (%)	65 ± 6	70 ± 4	68 ± 4

### Effect of brominating agent on yield of acetophenone derivatives

The effects of pyridine hydrobromide perbromide, NBS, and cupric bromide on the yield of 4-chlorophenone bromination were investigated. As shown in Table 5, pyridine hydrobromide perbromide exhibited the highest efficiency with a yield of 85% under identical conditions. The NBS reaction showed poor performance as only a small amount of products were generated after three hours, mostly consisting of unreacted starting materials. Copper bromide-mediated bromination resulted in a moderate yield (~ 60%).

**Table 5:** Effects of reaction time on yield of acetophenone derivatives

Brominating agent	pyridine hydrobromide perbromide	CuBr_2_	NBS
Yield (%)	85 ± 4	60 ± 6	-

“-” denotes an insufficient amount of product for separation and purification

### TLC monitored the reaction process

**Fig. 4:**
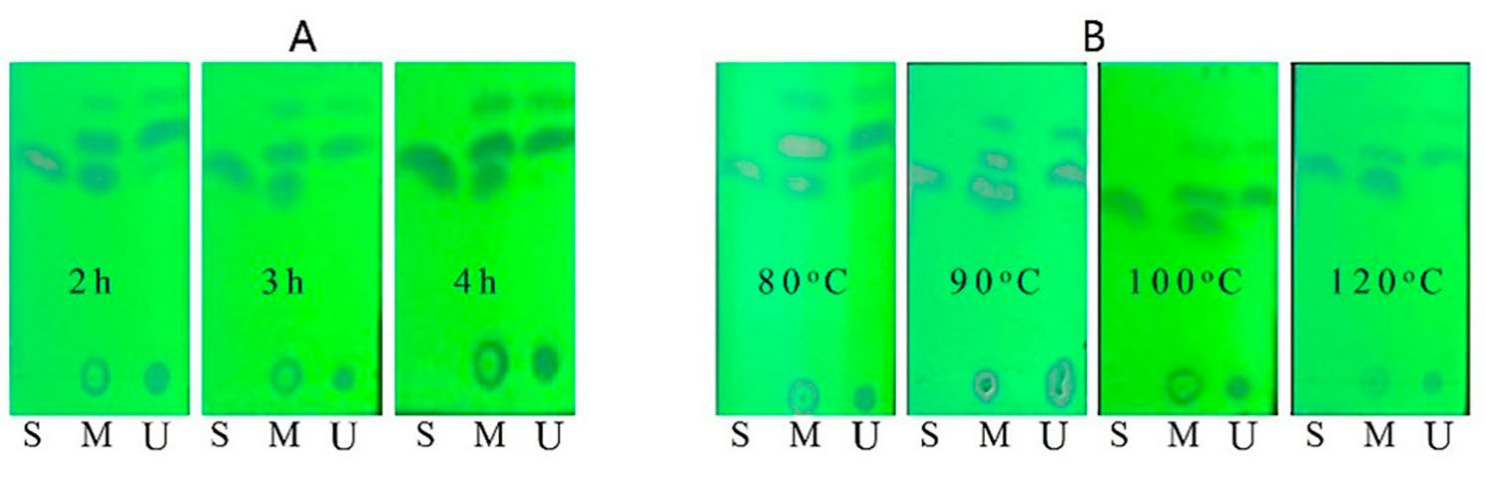
Effects of reaction time **(A)** and temperature **(B)** on bromination of 4-trifluoromethylacetophenone monitored by TLC. S represents starting material (4-trifluoromethylacetophenone), U represents unpurified product, and M represents their mixture

Thin layer chromatography (TLC) is a very effective means to monitor the progress of reactions in real time. Figure 4 shows the reaction of 4-trifluoromethylacetophenone with pyridine perbromide monitored by TLC under different reaction time (Fig. 4A) and temperature (Fig. 4B). The mixture of petroleum ether:ethyl acetate = 1:5 (v/v) was used as the development agent and observed under ultraviolet lamp (λ = 254 nm). As shown in Fig. 4A, the starting materials gradually decrease with the increase of time, and the starting materials basically disappear after 3 h of reaction. If the reaction time is increased to 4 h, the by-product increases significantly. Figure 4B shows the effect of different temperatures on bromination reactions. It can be seen that as the temperature rises, the starting material gradually decreases, and the temperature rises to 90 °C, and the starting material basically disappears. Therefore, the reaction of 4-trifluoromethylacetophenone with pyridine perbromide for 3 h at 90 °C can completely transform the starting material, and this reaction condition is suitable for application and promotion in undergraduate experimental teaching.

### Determination of melting point

The melting points of 6 products (2-bromo-4’-trifluoromethyl acetophenone, 2-bromo-4’-trifluoromethoxyacetophenone, 2-bromo-4’-chlorophenone, 2,4’-dibromoacetophenone, 2-bromo-4’-iodoacetophenone, 2-bromo-4’-phenylacetophenone) were determined and compared with the literature values (Table 6). The measured values were consistent with those in the literature.

**Table 6:** Determination and literature value of melting point of different acetophenone derivatives

Brominated Acetophenone Derivatives	Measurement Value	Literature Value
2-Bromo-4’-trifluoromethylacetophenone	46.7–49.3℃	47–50℃ [19]
2-Bromo-4’-trifluoromethoxyacetophenone	50.4–51.7℃	50-51.5℃ [20]
2-Bromo-4’-chlorophenone	95.3–97.6℃	96–98℃ [21]
2,4’-Dibromoacetophenone	108.6-112.4℃	109–111℃ [22]
2-Bromo-4’-iodoacetophenone	110.6-113.3℃	112–114℃ [23]
2-Bromo-4’-phenylacetophenone	122.8-124.6℃	123–125℃ [24]

### Characterization of bromoacetophenone derivatives

**Fig. 5:**
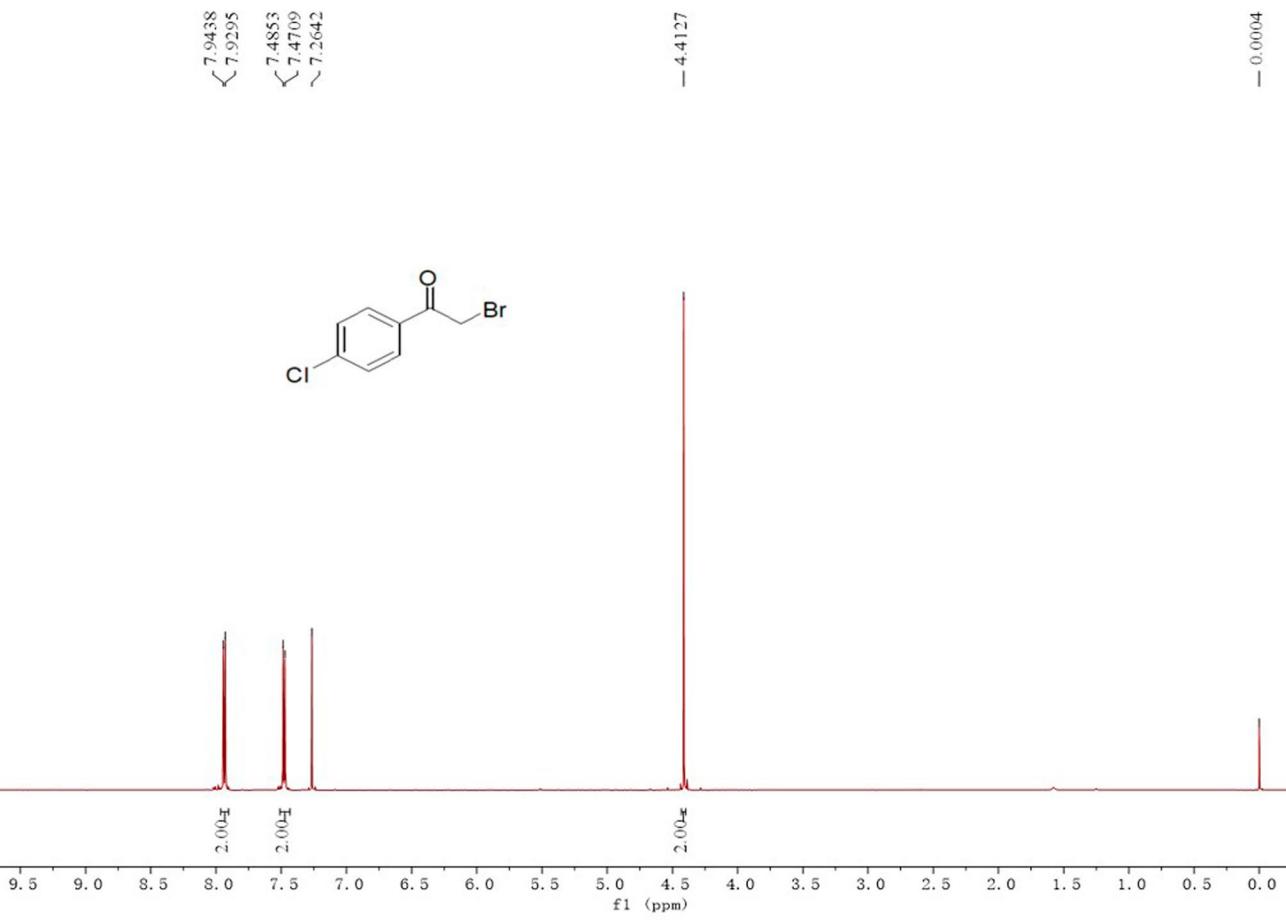
^1^H NMR spectra of 2-bromo-4’-chloroacetophenone

Six kinds of purified products were characterized by ^1^H NMR respectively. The data and spectra (The spectra of 2-bromo-4’-chlorophenone are depicted in Fig. 5, while the spectra of other compounds can be found in the supplementary materials) are as follows: ^1^H NMR (400 MHz, CDCl_3_): δ 7.94 (d, *J* = 5.72 Hz, 2 H), 7.48 (d, *J* = 5.76 Hz, 2 H), 4.41 (s, 2 H).

### Comparison of bromination reaction effects

In this experiment, 6 kinds of acetophenone derivatives were used to react with pyridine hydrobromide perbromide in acetic acid. This reaction has the advantages of cheap starting materials, high reaction yield, mild reaction conditions, simple operation and convenient post-treatment. It is suitable for undergraduate basic chemistry teaching experiment. Table 7 lists the reaction effects of 6 acetophenone derivatives substituted with brominated reagents under optimal reaction conditions (n_acetophenone derivative_:n_brominated reagent_ = 1.0:1.1, reaction temperature 90 °C, reaction time 3 h). The highest yield (90%) is achieved by bromination of 4-trifluoromethylacetophenone, but it is more expensive. Although the reaction yield of 4-chloroacetophenone is slightly lower than that of 4-trifluoromethylacetophenone (85%), it is cheaper. Therefore, the substitution reaction between 4-chloroacetophenone and pyridine hydrobromide perbromide is more suitable for undergraduate chemistry experiment teaching.

**Table 7:** Comparison of reaction effects of 6 acetophenone derivatives with pyridine hydrobromide

Acetophenone Derivatives	Yield	By-product	Cost
4-(Trifluoromethyl)acetophenone	90%	Less	Expensive
4-(Trifluoromethoxy)acetophenone	88%	More	Expensive
4-Chloroacetophenone	85%	Less	Cheap
4-Bromoacetophenone,	78%	More	Cheap
4-Iodoacetophenone	66%	More	Cheap
4-Phenylacetophenone	70%	More	Cheap

### Impact of innovative experiments on students

We examined the impact of an innovation experiment on three students who developed it, utilizing questionnaires (Table 8). Our investigation focused on the effects of their participation in the development experiment across five dimensions: scientific research proficiency, teamwork skills, problem analysis and solving abilities, as well as communication aptitude. The survey findings demonstrate a significant enhancement in all aforementioned aspects after four months of practice, thereby establishing a solid groundwork for future scientific research endeavors. Furthermore, through questionnaire-based assessments conducted among 18 tested students (Table 9), we highlight that the newly devised teaching experiment possesses several advantages including a balanced experimental principle, uncomplicated equipment requirements, feasible procedural steps and facile product purification processes. Consequently, this experiment is deemed suitable for application and promotion within undergraduate chemistry laboratory instruction.

**Table 8:** The impact of the innovation experiment on the three students

Impact effect	No effect	Little effect	Significant effect
Number of students			
Ability for scientific research	0	0	3
Ability to collaborate	0	0	3
Ability to analyze and solve problems	0	0	3
Ability to communicate	0	1	2
Consciousness of innovation	0	0	3

**Table 9:** The evaluation of 18 students on the new teaching experiment

Degree of difficulty	Easy	Moderate	Difficult
Number of students			
Experimental principle	3	14	1
Experimental device	12	5	1
Experimental procedure	14	2	2
Product purification	11	6	1

## Conclusion

Using scientific research methods, three undergraduate students conducted teaching experiments in organic chemistry to develop innovative approaches for promoting α-substitution reactions of acetophenone derivatives in undergraduate chemistry experiments. The feasibility of the experimental scheme was verified through experimental teaching, addressing the existing gap in this area. Participation in this innovation experiment significantly enhanced students’ communication skills, self-presentation abilities, problem analysis and solving capabilities. The experimental operations involved constructing heating devices, monitoring TLC (thin-layer chromatography), performing solvent extraction separations, purifying through recrystallization, and encompassed all fundamental steps of undergraduate chemistry experiments. This comprehensive approach played a crucial role in consolidating students’ basic experimental skills, enhancing their scientific literacy, fostering an innovative mindset among them and stimulating their interest in scientific research.

### Electronic supplementary material

Below is the link to the electronic supplementary material.

Supplementary Material 1

## Data Availability

No datasets were generated or analysed during the current study.
