# Supplementary material for: Application of α-bromination reaction on acetophenone derivatives in experimental teaching: a chemical innovation experiment engaging junior undergraduates
Source: BMC Chem. 2024 Feb 21;18(1):38. doi: 10.1186/s13065-024-01145-y (PMC10882791; doi:10.1186/s13065-024-01145-y)
Supplement: Supplementary file 1 — Supplementary Material 1 [file 13065_2024_1145_MOESM1_ESM.docx]

**Supporting Information**

**Application of α-Bromination Reaction on Acetophenone Derivatives in Experimental Teaching: A Chemical Innovation Experiment Engaging Junior Undergraduates**

**
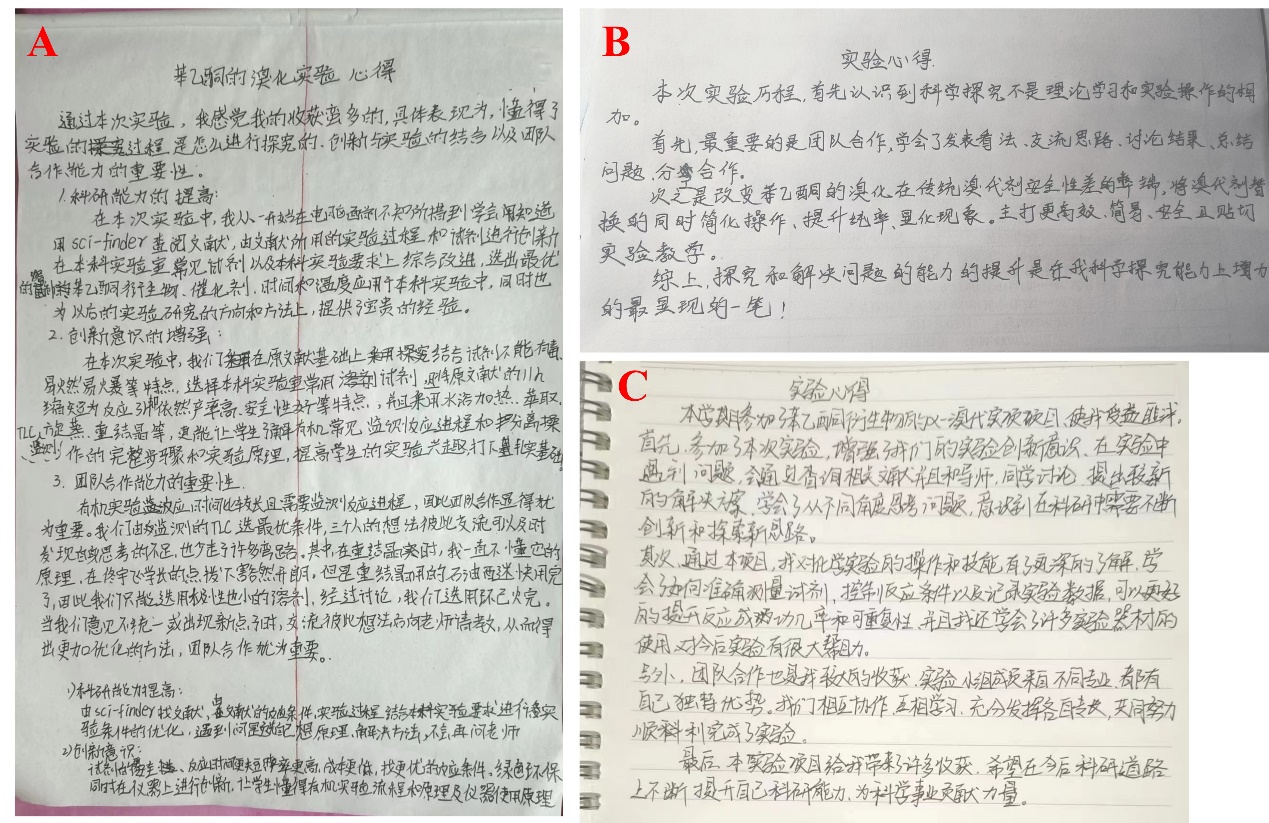
**

**Figure S1** The experimental experience of three students participating in the project


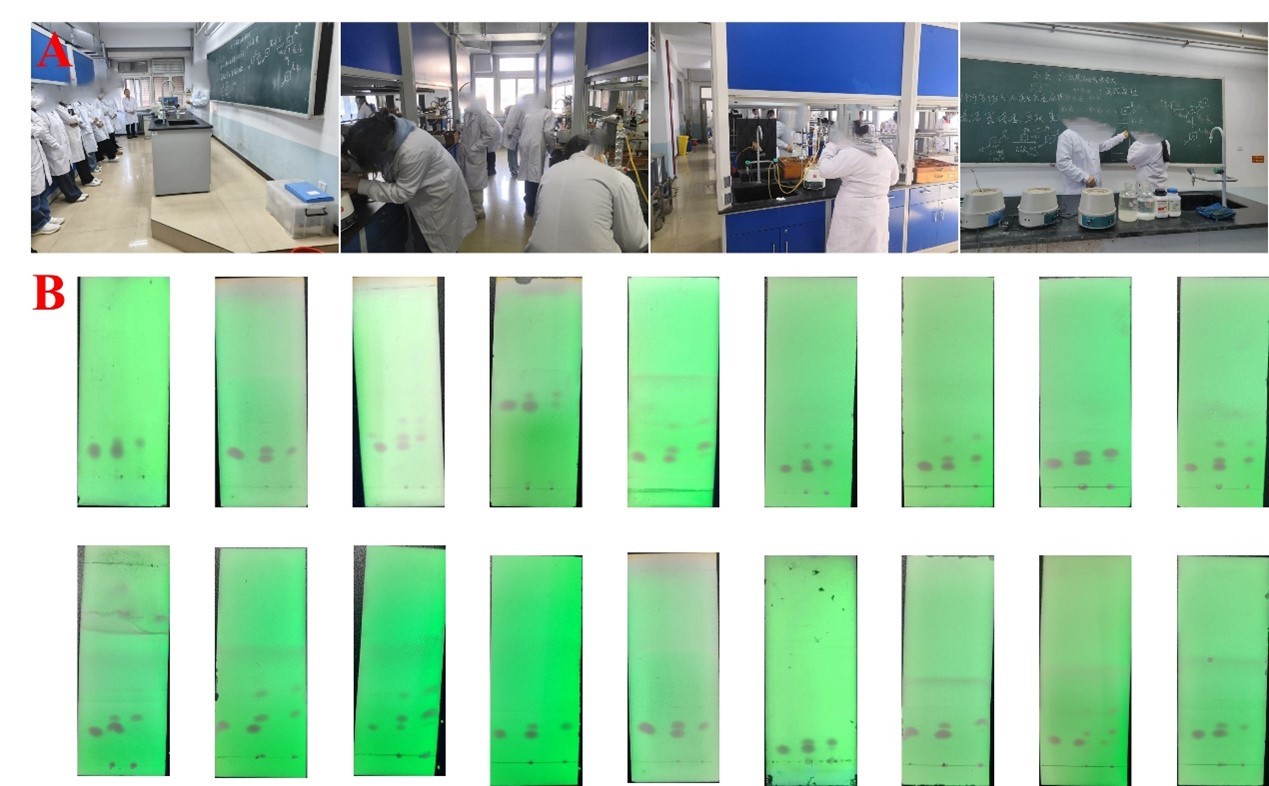


**Figure S2** Experiment teaching in chemistry laboratory (A) and TLC used by students to monitor reactions (B)

**Table S1** Experimental instruments

| **Experimental instruments** | **Manufacturers** |
| --- | --- |
| Rotary evaporator | Shanghai yarong biochemistry instrument factory |
| Electronic analytical balance | Ohaus Instruments(shanghai) Co., Ltd. |
| Ultraviolet analyzer | Shanghai Lichen Bangxi Instrument Technology Co., Ltd. |
| Vacuum drying oven | Shanghai Yiheng Scientific Instrument Co., Ltd |
| Magnetic heating stirrer | Shanghai Lichen Bangxi Instrument Technology Co., Ltd. |

**Table S2** Reagents and materials

| **Reagents** | **Reagent purity** | **Suppliers** |
| --- | --- | --- |
| 4-Trifluoromethylacetophenone | Analytical reagent | Beijing Ouhe Technology Co., Ltd. |
| 4-Trifluoromethoxyacetophenone | Analytical reagent | Beijing Ouhe Technology Co., Ltd. |
| 4-Chloroacetophenone | Analytical reagent | Beijing Ouhe Technology Co., Ltd. |
| 4-Bromoacetophenone, | Analytical reagent | Beijing Ouhe Technology Co., Ltd. |
| 4-Iodoacetophenone | Analytical reagent | Beijing Ouhe Technology Co., Ltd. |
| 4-Phenylacetophenone | Analytical reagent | Beijing Ouhe Technology Co., Ltd. |
| pyridine hydrobromide perbromide | Analytical reagent | Beijing Ouhe Technology Co., Ltd. |
| Ethyl acetate | Analytical reagent | Tianjin Fuyu Chemical Agent Co., Ltd. |
| Petroleum ether | Analytical reagent | Tianjin Fuyu Chemical Agent Co., Ltd. |
| Acetic acid | Analytical reagent | Tianjin Yongda Chemical Agent Co., Ltd. |

**Experimental Scheme**

**I Learning Objectives**

1. Study the principle and method of preparing 2-bromo-4'-chloroacetophenone by substitution reaction of 4-chloroacetophenone with pyridine hydrobromide perbromide.

2. Master the experimental operation of stirring, reflux, extraction and recrystallization.

**Ⅱ Principles**

α-Bromoacetophenone derivatives serve as crucial intermediates in organic synthesis, finding extensive applications in the production of pharmaceuticals, pesticides, and other chemicals. For instance, α-bromoacetophenone is a significant intermediate for non-steroidal anti-inflammatory drug aryl propionate, while p-methoxy-α-bromoacetophenone acts as the primary intermediate in synthesizing the estrogenic drug raloxifene. O-chloro-alpha-bromoacetophenone plays a vital role as an intermediate for clorprenaline. These derivatives are typically obtained through α-bromination of acetophenone derivatives using brominating agents. Commonly used bromination reagents include liquid bromine, n-bromosuccinimide (NBS), and copper bromide. Liquid bromine exhibits high toxicity, strong corrosiveness, low reaction selectivity, environmental pollution risks and poor safety measures. NBS and copper bromide offer good selectivity and high safety levels but are expensive. Pyridine hydrobromide perbromide (Py•HBr_3_) offers notable advantages in terms of its high safety profile, exceptional and cost-effectiveness. The bromination reaction equation of 4-chloroacetophenone with pyridine hydrobromide perbromide is depicted in the figure below.

The reaction mechanism of α-bromination of 4-chloroacetophenone is illustrated in the figure below. Under acidic conditions, 4-chloroacetophenone **1** undergoes protonation to yield protonated carbonyl compound **2**. Subsequently, bromine ions attack the hydrogen atoms on the alpha carbon of compound **2**, leading to the formation of enolate product **3** after debromination. Compound **3** then undergoes a nucleophilic attack by the electron on positively charged bromine ions, resulting in the formation of compound **4** through the creation of a carbon-oxygen double bond with the lone electron pair on hydroxyl oxygen. The rate-determining step for this reaction is identified as the second step involving enol form **3**. In cases where an electron-donating group is attached to the alpha carbon of the carbonyl compound, it becomes challenging for protons to leave and consequently slows down the reaction rate. Conversely, when an electron-withdrawing group is present at this position, protons are more easily released and thus accelerate the reaction rate accordingly. Similarly, attachment of an electron-withdrawing group to a benzene ring also facilitates α-bromination reactions in acetophenone derivatives.

**III Apparatus and Reagents**

Rotary evaporator, electronic analytical balance, ultraviolet analyzer, vacuum drying oven, magnetic heating stirrer, Buchner funnel, separatory funnel, suction flask, round-bottom flask (50 mL), Allihn condenser, and calcium chloride tube. The reaction device is shown in the figure below.

4-Chloroacetophenone, Py•HBr_3_, ethyl acetate, petroleum ether (60-90 ^o^C) and acetic acid.


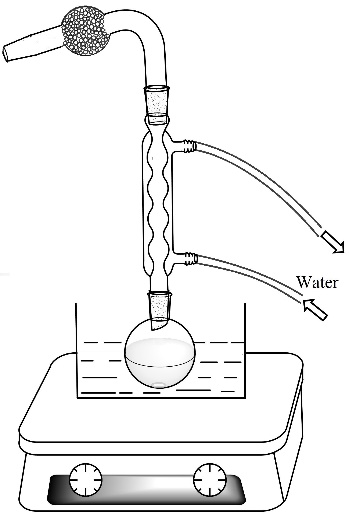


**IV Procedures**

4-Chloroacetophenone (0.77 g, 5.0 mmol), pyridine hydrobromide perbromide (1.76 g, 5.5 mmol), and acetic acid (20 mL) is combined in a 50 mL round-bottom flask equipped with a condensing tube, and the reaction mixture is stirred at 90 ^o^C. The reaction progress is monitored via thin layer chromatography (TLC), and the depletion of the starting material is observed at a time point of 3 hours post-reaction. Upon completion of the reaction, the resulting mixture is poured into an ice water bath (50 mL) and extracted twice with ethyl acetate (20 mL × 2). Organic phase is sequentially washed with saturated sodium carbonate solution (30 mL) and saturated saline solution (30 mL). After drying over anhydrous sodium sulfate, the solvent is removed under reduced pressure using a rotary evaporator to afford crude solid product which undergo recrystallization from petroleum ether (-4 ^o^C). The resulting orange solid product is confirmed by ^1^H NMR spectroscopy. The melting point ranges from 95.3 to 97.6 ^o^C.

**V Notes**

1.The melting point of anhydrous acetic acid is 16.6 ^o^C, and if it is a solid before use, it can be measured after melting with warm water. Acetic acid is more corrosive to the skin, such as accidentally on the skin, should be immediately washed with soapy water and water.

2. The reaction temperature is precisely maintained at approximately 90 °C, as exceeding 100 °C would result in a significant formation of dibromine substitution products.

**^1^H NMR spectra of α-bromoacetophenone derivatives**
